# Hospital and Institutional News

**Published:** 1913-11-29

**Authors:** 


### HOSPITAL AND INSTITUTIONAL NEWS.

### SOME AMUSEMENT AND A LITTLE MONEY.

The Hospital this year, as usual, will publish a Special Appeal Number, exhibiting what the spirit of personal service achieves for those who give and those who receive. In order to bring home to the public what is the spirit of personal service within the institutions themselves, we offer five prizes of two guineas for the best illustrated account or one guinea for the best account without illustrations of "How to Spend Christmas in a Hospital," from the five points of view of the Resident Medical Staff, the Secretariat, Matron, Sister, or Nurse, competitors in each group to represent one point of view only. Every type of institution is included, and *in addition* a special prize of three guineas will be awarded to the best account by the representative of any group, with illustrations. Any writer therefore may have a chance of winning five guineas, and the essays will reveal how few or how many institutional workers there are in the country alive to the spirit of personal service in themselves, and to a conscious understanding of it as the driving force in institutional life. **Prize essays must reach us not later than December 11, 1913.** They should be written plainly (preferably typed) on one side of the paper only, contain not more than 1,000 words, and be accompanied by the coupon from our current issue. The Editor's decision and awards are to be final and unquestioned. The prize essays will be published in the Christmas Number.

### THE SUPERINTENDENTSHIP AT ST. GEORGE'S HOSPITAL.

A few weeks ago, it will be remembered, we commented upon an extraordinary proposal, which was said to be entertained by some of the managers of St. George's Hospital, for the appointment of a paid treasurer (The Hospital, October 11, 1913; p. 27). Our criticisms seem to have sufficed to extinguish this most undesirable scheme, for nothing has come of it. Other developments have, however, taken place quite lately which will astonish the hospital world almost as much as a paid treasurership. To understand the present position it is necessary to go back some seven years, when the non-resident secretary-superintendent, Mr. Todd, resigned after many years of faithful service. At that time the officer responsible for discipline and supervision after office hours was the resident medical officer, Dr. F. J. Marshall, who had for thirty years and more worked harmoniously with the secretary. Shortly after these two officials retired a change of system was introduced. A resident medical superintendent was appointed, and the purely secretarial part of Mr. Todd's duties was allotted to his second in command, Mr. H. Wingrove, who was designated secretary to the house committee. This arrangement worked fairly well, thanks to the tact and ability of Dr. G. E. Friend, who resigned the post of medical superintendent this year on his appointment as medical officer to Charterhouse School.

### AN APPOINTMENT "IN CAMERA"?

Now, it is understood, a return is to be made at St. George's Hospital to a system resembling that which was abandoned six or seven years ago. Mr. Wingrove retires after many years spent in the service of the hospital, the last six of them as secretary to the house committee. Once again the lay secretary is to be superintendent also, and the resident medical officer is to have none of the administrative functions which Dr. Friend discharged. The honorary treasurer of the hospital, Mr. A. W. West, is resigning that office; and is to be appointed, if our information is correct, to the post of paid non-resident secretary-superintendent, without any of the usual advertisement of the vacancy. If this arrangement really is contemplated, or already concluded, it is at least preferable to the paid treasurership of which we expressed our disapproval seven weeks ago. But with the projected amalgamation and removal schemes looming ahead, St. George's will need all the support from outside which it can get. Such support will depend in large measure on the reputation of the managers for good sense and business ability. Without attaching any importance to vague rumours about a hitch in the negotiations for the sale of the present site, the protracted dispute with the King's Fund has not increased public confidence in the board of St. George's, and such a strange appointment as the one referred to could not do much to restore it. Mr. West is an architect, not a hospital administrator. Can the governors fail to insist that St. George's Hospital to succeed must secure the ablest and most knowledgeable administrator as its secretary-superintendent? His first duty will be to end the troubles caused by women governors or the relatives of governors and other interferers with the internal administration of this hospital.

### THE NEW BUILDINGS AT THE RADCLIFFE INFIRMARY, OXFORD.

The opening of the new buildings of the Radcliffe Infirmary, Oxford, by Earl Curzon of Kedleston, Chancellor of the University of Oxford, on Wednesday, November 26, may prove memorable in the history of our voluntary hospitals. It marks a new departure of supreme importance, for, thanks to Sir William Osler's initiative and suggestion, the Radcliffe Infirmary ought to become the first county centre to which all medical practitioners within a radius of twenty or twenty-five miles should habitually resort for graduate instruction and up-to-date knowledge. It is not possible, owing to the time we go to press, to give a full report this week. We may mention, however, that the ceremony was a great success, that Lord Curzon proved a very amusing as well as an able speaker, that Mr. Cronshaw surpassed his former record, which is exceptionally high, as a host, and that Sir William Osler and the Mayor of Oxford by their speeches added to the interest and charm of a remarkable occasion. It is remarkable in the sense that the late Lord

Salisbury, as Chancellor, in presiding in 1893 at a meeting at the Sheldonian Theatre in support of a scheme for reorganising the Radcliffe Infirmary, gave a forecast of what would constitute its ideal equipment "with all that science and practice can furnish to make it most efficacious for the relief of suffering." On Wednesday—*i.e.*, twenty years afterwards—his successor, Lord Curzon, crowned the completion of the work and testified to the realisation of Lord Salisbury's ideal by opening the new buildings which finish the work of reconstruction. We hope to give next week a full report of the proceedings and to show Sir Henry Acland's share in the scientific development of the Radcliffe Infirmary.

### FURTHER MEETINGS OF MENTAL MEDICAL OFFICERS' ASSOCIATION.

In view of the meeting held by the new Association of Assistant Medical Officers of Public Mental Hospitals in the Midlands, which we noted last week, it is interesting to recall that the Durham County Mental Hospital as lately as October last circularised its fellow-institutions suggesting a conference to consider how best to improve the conditions of the service. Dr. F. J. Stuart, the senior assistant medical officer of Northampton County Mental Hospital, then started a correspondence in the medical press, and is now acting as provisional secretary. The new Association was formally inaugurated by resolution at the Birmingham meeting, and from thirty mental hospitals approval was expressed with the object of the meeting. A guarantee fund of £100 has been raised, and a provisional committee has been appointed. In the meantime other meetings are arranged for, of which the first is to be held at Manchester on December 3, when representatives of the Northern public mental hospital medical staffs will meet officers of the Association. On Thursday, December 4, representatives of the Association will meet at Bristol officers of the South Wales and West of England mental hospitals similarly to discuss the future of the Association's work.

### GUARDIANS AND THE TEACHING OF ANATOMY.

The well-known scarcity of subjects for dissection and the teaching of operations led to an urgent request for legislation by Sir Victor Horsley at the annual meeting of the Royal College of Surgeons. The annual report stated that consultations with the licensed teachers of anatomy had been held, and that the President, with a deputation, had attended at the Home Office. In reply to a remark of Sir Victor Horsley's that the only way in which anything could be done was by legislation, Mr. Makins, who was in the chair in the absence of the President, said that the Home Office had met them kindly, but had been unable to suggest any legislation. Medical members of Parliament very much disliked the idea of bringing the matter forward. In this case we fear that the Parliamentary atmosphere must have had a deteriorating effect upon the scientific conscience, but it would have been more fruitful if Sir Victor Horsley had at least provided the Home Office with a basis of discussion by suggesting the lines which, in his view, legislation ought to take. Importation must either be made more easy or the supply of subjects in this country must be increased by giving Guardians facilities not yet provided by the law.

### THE TITLE "DOCTOR."

Consecrated by public usurpation to all engaged in the practice of medicine or surgery, the title "doctor" has been forced on practitioners, with or without the degree of "M.D.," in spite of themselves. This, however, is now to be winked at officially, so far as the practitioners' use of it, by the two Colleges concerned. The report of the Council of the Royal College of Surgeons, referred to by Mr. G. H. Makins, C.B., at the annual meeting, notes that the Royal College of Physicians has withdrawn its former prohibition to the use of the title by licentiates, and is, therefore, now on a par in practice with the Royal College of Surgeons. Thus have official bodies been compelled to bow to an inveterate public habit. Indeed, the latent irritation which is felt by many laymen when Dr. Smith or Dr. Brown turns out to be a doctor of science, or divinity, or literature, instead of a medical man, may yet affect some day a change even in these titles.

### HOSPITAL COLLECTING A MINER'S HOBBY.

An interesting ceremony has occurred at Walsall Hospital with the presentation of a silver medal to Mr. John Holmes for his work in the district for the hospital. As an example of undiminished personal service over a long period of years Mr. Holmes' annual collection is perhaps unrivalled. Practically all his spare time—and as a working miner this is necessarily limited—is spent in collecting for the hospital. By this time he has received quite a number of medals in recognition of what is his life work, and in making this latest presentation Mr. F. J. Cotterell, the chairman, stated that the sum total collected by Mr. Holmes now amounted to over £322. Among the many varieties of "Relaxations and Hobbies" on which we have published articles hospital collecting has never been described, but the above instance of enthusiasm in pursuit of it is sufficient to show that it can be as full of interest as any other.

### THE MEDICAL MAYORS OF BATH.

Unusual interest attached to the meeting of the managing board of the Bath Royal United Hospital last week, from the fact that the new Mayor, Dr. Preston King, attended for the first time as president of the institution. In welcoming him the chairman, Mr. Egbert Lewis, referred to the fact that on two previous occasions a member of the hospital's staff had been Mayor of Bath—namely, when Dr. Falconer held the post in 1857-58, and Mr. Freeman in 1888. Dr. Preston King, however, has the satisfaction of establishing a new record, since this year is the first occasion on which the same man has been Mayor, president of the hospital, and a member of the staff. The new Mayor, already known to the city as a member of the medical profession and as an author of an "account" of the Bath waters, is likely to identify more closely than ever the prosperity of the spa with the medical mayors.

### TECHNICAL ASSAULT BY TEETH EXTRACTOR.

At Bow County Court this week £15 damages and costs were awarded against a South Woodford resident for committing a technical assault by administering cocaine during the extraction of the plaintiff's teeth against the expressed wish of the plaintiff. The defence denied the request. Mr. Judge Smyly was of opinion that it had, in fact, been made, and that this constituted a technical assault. The defendant's name does not appear in the Dental Register, and it would seem that "a teeth extractor" has been unexpectedly caught napping.

### WESTMINSTER HOSPITAL SITE PROPOSAL.

In the course of a paper on the new Central Hall, Westminster, read before the Royal Institute of British Architects last week, Mr. H. V. Lanchester made some ingenious suggestions with regard to the manipulation of Westminster Hospital site, so as to improve Broad Sanctuary without injustice to any of the parties concerned. The line of the hospital, he said, should be set back, as Broad Sanctuary was not nearly broad enough for ceremonial occasions. In order to do this he pointed out that the site at the back of the hospital was occupied by the Stationery Office, which itself is to be removed shortly to Stamford Street. This land is not of exceptional value, he remarked, since it has no frontage on an important street, and the Government therefore ought to give about a third of its area to Westminster Hospital in exchange for a corresponding area in Broad Sanctuary. By this scheme, he contended, the nation would get the dignified space which it needed, the hospital would be as well off with the exchange, and the Government would give up land about half the value in proportion to that now occupied by the institution. This most ingenious scheme is certainly worth while pressing upon the Government's attention, for, providing that no other facts have been overlooked by Mr. Lanchester, the State has an opportunity of doing something which is badly needed at no cost to itself. Unfortunately simplicity, need, and opportunity prove often as little alluring to English Governments as opposition, expense, and complexity. What have the Westminster Hospital authorities and Mr. Quennell to say to Mr. Lanchester's proposal?

### LATE ADMISSIONS AT LEWISHAM INFIRMARY.

The difficulties of an infirmary medical staff, where patients are admitted to an institution on the advice of private practitioners, are exemplified in the case of Lewisham Infirmary. A large number of patients are admitted, who pay for the whole cost of their maintenance, and these are generally sent by the local doctors. It has been found that the latter are in the habit of sending patients into the infirmary late at night, probably after seeing them during their evening consultation hours. Much disorganisation has resulted through the late admission of these patients. To remove the confusion that has arisen the Board has decided to send a circular-letter to the medical men in the district, drawing their special attention to the fact that there is no special night medical staff to attend to urgent cases. They are desired to send their patients not later than 4 o'clock in the afternoon.

### AN INSPECTOR ON THE WORKHOUSE CHILD.

Mr. H. R. Williams, Local Government Board inspector, gave a very direct address to the Wrexham Board of Guardians last week, in which, after praising them for the economies effected in the cost of maintenance, and suggesting tnat the higher cost of out-door relief was due to the more adequate relief now wisely given by the Guardians to widows with children, he put in a strong plea for the workhouse child. Guardians, he said, had no possible excuse for having children in the workhouse at the present time. Decent food and clothing notwithstanding, the workhouse boy and girl, he affirmed, carried a different atmosphere about with them from that of children brought up outside. The best method, in his opinion, was the boarding-out of the children under proper conditions. The scattered home had advantages, but that entirely depended upon the foster-mother, while the cottage home was another method. The real difficulty is that the child in the workhouse cannot be classified and kept apart from its other inmates. The institutional atmosphere has many advantages, besides a few drawbacks, for the growing child, but in order that the advantages may preponderate it must be an institution in which many children are accommodated. It is the mixed ages, as much as the undesirable influences of certain adults, which make the workhouse no fit place for a child.

### WHAT IS A MENTAL HOSPITAL?

The improvements in the institutional treatment of patients suffering from mental diseases are making the term mental hospital a generic one to describe all institutions in which the insane or mentally deficient are confined. But the two words have in the technical sense a more precise meaning, which is worth notice, however much we may commend their improvement on "asylum" and similar avoidable terms. Under the Lunacy Acts three types of institutions are recognised: the County and Borough Mental Hospitals (the asylums of old), Registered Hospitals, and Licensed Houses. The county and borough institutions are rate-supported, and intended for the Poor-Law class of patient; while the registered hospitals and the licensed houses exist for paying patients, have no rate-aid, and sometimes no endowment. Now that the phrase mental hospital is becoming a generic term it is worth remembering, and impressing on laymen, that each of the three species which compose it is coming to use the title, for the attempt made some years ago to capture the word sanatorium by institutions for the care of the insane was rendered unsuccessful by the anti-tuberculosis movement, in which the public gave prescriptive rights in this word to the new chest hospitals. To-day, however, the mental hospital is becoming the property of the new institutions for unimprovables, the word colony being increasingly used for those where the ability of the majority is rendered capable of useful work.

### THE CITY PUBLIC HEALTH DEPARTMENT.

Mr. Henry Montague Bates, principal clerk in the Public Health Department of the City, has resigned his appointment owing to a recent accident. It was in 1875 that Mr. Bates first entered the service of the Corporation, where he was assistant clerk and later clerk to the City Commission of Sewers. In 1898 the Commission and the Corporation amalgamated, and he was appointed principal clerk at a salary of £1,800 a year. In any case Mr. Bates would have reached the age-limit in March next, and he now retires on a pension, estimated on the two-thirds scale, which is granted under the City of London Sewers Act. On the retirement of Dr. Collingridge from the post we published a striking interview with him on the amazing growth and complexity of the Public Health work of the City. Readers of that article will gain some idea of the changes which have taken place in Mr. Bates' time.

### THE NEUROLOGIST AT CHARING CROSS.

The vacancy on the honorary medical staff at Charing Cross Hospital created by the resignation of Dr. F. W. Mott has been filled by the promotion of Dr. W. Cecil Bosanquet to the office of full physician. For the vacancy thus made among the out-patient staff there were several strong candidates. The selection committee last week unanimously nominated Dr. Gordon M. Holmes for the post of assistant physician to the hospital. Dr. Gordon Holmes has already made a reputation for himself in the world of neurology; his researches and published papers have been both important and numerous. He is at present assistant physician at the National Hospital for the Paralysed and Epileptic, Queen Square, at which institution he was formerly the resident medical officer, and also held a similar appointment at the Seamen's Hospital, Greenwich. Dr. Holmes qualified in 1898 at Dublin University, where in the previous year he had gained the gold medal in the B.A. Examination. He has since done a great deal of neurological research in Germany, particularly in the Senckenberg Laboratory at Frankfurt. In electing Dr. Holmes to a position made vacant by the retirement of one great neurologist the committee of Charing Cross Hospital have been alive to the need for maintaining the high prestige of this appointment.

### THE POOR-LAW CONFERENCE.

Mr. G. E. Lloyd Baker presided over the annual meeting of the Central Committee of Poor Law Conferences last week, at which the announcement was made that the London Boards of Guardians had decided to hold a separate annual conference in future to consider questions which are not interesting to rural unions. The London Boards, therefore, will cease to hold their joint meetings with the South-Eastern Conference. Lord Richard Cavendish was elected chairman of the committee in succession to Mr. Lloyd Baker, and Lord Methuen as president of the coming Central Poor Law Conference, which will be held next February in London. The overlapping of old-age pensions, national insurance, and the Poor Law, housing, and, of course, the Mental Deficiency Act are down for consideration at the conference.

### LIMITS OF MEDICAL REFEREES: PANEL DOCTOR'S PROTEST.

At a meeting of the Birkenhead Insurance Committee, last week, Dr. John Grimshaw brought before it the question whether an approved society can override, through their medical referee, the certificate of a panel doctor that a patient is unfit for work. In a case which Dr. Grimshaw described in detail, one of his patients certified as unfit by him three days later returned with the word "fit" substituted on his sick benefit sheet, initialled by the society's medical referee. Dr. Grimshaw thereupon inquired of the Insurance Commissioners whether the medical referee had any legal right to examine a patient in receipt of medical benefit without intimating his intention to the panel doctor, and to cancel his signature without consulting with him in any way. Consultants' opinions confirmed the original pronouncement of unfitness. Eventually the Commissioners replied that "the question whether incapacity exists is, in the first instance, for the society to decide," while a member has "a right of appeal to the tribunal appointed under the rules of his society for the settlement of disputes," and, in the last resort, to the Commissioners. In spite of consultants' opinion the society, Dr. Grimshaw continued, refused to reply to his communications and to pay the sick benefit, refusals which were communicated by him to the Commissioners. The Poor Man's Lawyer of the Charity Organisation Society was secured by the doctor to take up the case, and he thereupon moved that the question of the relation of medical referee, insured person, and panel practitioner be referred to the Medical Service Sub-Committee for consideration and report, and eventually this was accepted, except that Medical Benefit Sub-Committee was substituted. Dr. Grimshaw was well advised and courageous to insist upon the limits and regulations imposed upon medical referees, and the result of the appeal will be awaited by us with great interest.

### HOUSING AS PREVENTIVE MEDICINE.

The social value of the school clinic and the consumption sanatoria, is not limited to local work, nor to the example which is thus set to those within their sphere of influence. The further we press medicine back into the sphere of prevention, the further back we find the need for prevention to be. Thus at the Conference on Housing organised by the Workmen's National Housing Council last week, and presided over by Mr. C. W. Bowerman, M.P., Alderman R. Morley, of Halifax, said that, instead of attacking sweating, bad food, and bad houses, expensive sanatoria were built to cure the people whom these evils manufactured. Instead of attacking the housing question school clinics were set up. This, of course, is the old story, but the fact remains that symptoms always prove more attractive than causes, because they are easier to recognise. All that can be said to palliate Alderman Morley's argument is that the recurrent, standing cost of institutional treatment is a good way of fixing attention on the expensiveness of a method which only arrives to aid after the mischief has been suffered without protest.

### DUBLIN MEDICAL OFFICER ON HOUSING.

THE Local Government Board inquiry into the housing of the working classes in Dublin has been the occasion of some interesting statements by Sir Charles Cameron, chief medical officer of health for the city. In reply to Mr. O'Connor, the chairman, Sir Charles said that many persons rented tenement houses and sublet them to tenants from whom the Corporation could not recover costs of repair. He knew that £2,000 had been spent on sanitary improvements by the Corporation some years ago, and attempts to recover it led only to further loss. He always hesitated to condemn areas as unfit for occupation because there was no proper alternative accommodation for those who would be dispossessed by drastic action; and the fact that one or two members of the City Council were property owners had never influenced him. From a hygienic point of view the tenement cannot be justified, but, short of municipal provision on a scale altogether out of practical politics at present, it will probably survive as perhaps the only way of extracting a profit from providing "housing" for the people, who are, in point of fact, too poor to pay for proper housing at all.

### PRIVATE SANATORIA AND INSURANCE PATIENTS.

THE history of private sanatoria in this country has been a chequered one. They began to be established about fourteen years ago, when the hygienic dietetic treatment of consumption was demonstrating its position as a real advance in phthisic therapy. The South African War produced quite a little crop of well-to-do patients, and for this and other reasons the supply of institutions before long exceeded the demand for them. Then competition thinned their ranks, and for a good few years only half a dozen or so were really successful, another factor contributing to this result being the establishment of sanatoria partly supported by charity which could afford to charge low fees. The hastily enacted Insurance Act (it is an ill wind, one remembers) did something to change this condition of affairs. An institution of the kind within our own knowledge, which had jogged along with only seven patients, became approved by a Government medical inspector, extended its wooden bounds, and soon was accommodating forty. But the proprietor added that he did not expect the boom to last, and indeed, as the approval of all such places is only stated to last until 1914, it is very probable that he was right.

### SANATORIA AND SITE VALUES.

Now Insurance patients being, generally speaking, of a different social class from the inmates formerly provided for, it is obvious that a neighbourhood near London which had found nothing to object to in a few well-dressed consumptives being housed in its district, might object to a much larger number not so taking to the eye. This seems to have been actually the case at Telford in Surrey. No complaints of misconduct were made—these mostly concern new institutions adapted as sanatoria and under inexperienced management—but the presence of the patients on the public roads and in the village shops was cited as dangerous to the local inhabitants, and a medical officer of health supported the memorial, adding significantly that the residential future of the place was threatened. It cannot be too widely known that well-conducted sanatorium patients are a danger to no one. Even in Davos, in Switzerland, an extreme case, tuberculosis among the natives has not increased at all since the village became a large consumption resort. However, it will be well, on other grounds, when 100-bed county sanatoria, with land attached for employment of patients and ex-patients, have been set going, and when this "boarding-out" system ceases. If a house and a proper salary are offered, there will be a choice available for the position of superintendent. But on the face of what we read in the *Times* about this memorial, there was no case for medical backing.

### PHARMACEUTICAL CHANGES AT THE UNIVERSITY COLLEGE HOSPITAL.

THE dispensary at the University College Hospital is about to lose its two well-known officers, Mr. Reginald R. Bennett, B.Sc., F.I.C., Ph.C., and Mr. T. E. Tawell, Ph.C., who will shortly retire. Both these gentlemen are relinquishing public pharmacy for, we suppose, the more remunerative sphere of commerce. Mr. Bennett goes to join the staff of the British Drug Houses, Ltd., and Mr. Tawell to become secretary and head of the sundries department of another well-known firm. Mr. Bennett is a prominent figure in pharmacy as an honorary secretary of the British Pharmaceutical Conference, which office he holds jointly with Mr. H. Finnemore, pharmacist at Guy's Hospital. Mr. Tawell has added to the literature concerning salvarsan, and has made some useful suggestions in regard to the pharmaceutical manipulation of that product.

### A CORRUGATED IRON HOSPITAL.

THE opening of the new hospital at Thornton, Fife, N.B., is interesting as providing one of the few examples of temporary structures in hospital building. Owing to the position of the site being close to a mining field, with the ever-approaching chance of minerals being worked under as well as close to the building, it was decided to form the additions and improvements in corrugated iron, or such impermanent material. A pavilion of 160 feet long, with a branch extending 24 feet, contains three wards: one general ward of sixteen beds, a smaller ward of eight beds, and a ward for phthisis cases with six beds, making a total of thirty. The women's wing contains accommodation for twenty-one. The architects, James Gillespie and Scott, have had the double task of improving the old buildings and constructing new ones of a temporary character, and this curious effect of circumstances, together with the peculiar position of the site, singles out this institution as perhaps unique in the conditions of its existence.

### A SANATORIA "BURDETT."

From time to time writers on tuberculosis in our columns insist on the approaching recognition of tuberculosis as a specialty, and now, as a practical sign that their hopes are being fulfilled, we receive the first issue of "The Tuberculosis Year Book and Sanatoria Annual," edited by Dr. T. N. Kelynack, who also edits a monthly devoted to tuberculosis. The new annual consists of a directory of sanatoria, homes, preventive associations, etc., together with a series of signed communications dealing with various problems, official duties, schemes, and information from other countries connected with the disease. Each sanatorium mentioned, with few exceptions—and we still doubt, so rapidly are sanatoria growing, if the list is complete—receives a descriptive account of its aims, terms of admission, and principal officers, signed by one of the responsible officials. The annual is illustrated and published by John Bale, Sons and Danielsson, Ltd., Great Titchfield Street, W., price 7s. 6d. net. Dr. Kelynack has made a useful attempt to amass the information needed in a new annual of this kind, and when it has been longer in existence the original communications will probably be strengthened by statistical chapters of information on the lines which have made Burdett's "Hospitals and Charities" the classic which it is. The sanatoria annual, however, is a valuable step on the road.

### CLERGY PENSIONS AND USELESS APPEALS.

The scope of our work covers an interest in all philanthropic enterprise. Year after year at this season we receive practically an identical set of papers from the managers of the Clergy Pensions Institution asking for contributions. Each year we have hoped that more business-like and up-to-date methods might be pursued in the drafting of these appeals with the view of adding force and attractiveness to them and a saving in the cost. Our hope has proved vain, however, and we are thus moved to point out that, although the scheme of the Clergy Pensions Institution appears to be based on self-help supplemented by benefactions, the nature of the actual scheme has never been sent us, and it is impossible for the recipient of an appeal to judge whether it is sound financially or even one which, apart from the declared object, calls for support from men of business. The papers declare "the expenses of management are nil, being defrayed by the business sections, and represent an unusually moderate percentage of the income as shown in the annual report." Is it not time that the co-operation of a few men of business and experience was secured to help Clergy Pensions to become, as they might easily be made, adequate to meet the claims and necessities of all by whom they may be needed? The sooner the present type of appeal is given up and an attractive, informative, terse and business-like document substituted for it, the better. We have no knowledge on the point, but we imagine the present papers, however widely they may be circulated, are not calculated to do much if any more than pay the cost of their printing and circulation. The thousands of pounds similarly thrown away on useless appeal literature grow year by year in number. Are there fewer brains available than formerly in this country, or what is the underlying cause of such annual fooling?

### NEW WING AT MELBOURNE CHILDREN'S HOSPITAL.

At the Melbourne Children's Hospital in August the Governor-General, accompanied by his children, the Hon. Judith and the Hon. Thomas Denman (who laid the foundation-stone), opened the completed new addition, called the Edward Wilson Surgical Pavilion, before a large gathering. The Sydney Women's Hospital, which was very much in debt, has been the object of an especial effort by the women of that city. Over £5,000 was collected privately, and the Government gives £1 for £1 on £3,500 of the sum, so that about £9,000 will be realised, and the hospital put on a sound financial basis.

### THIS WEEK'S DRUG MARKET.

This has again been a quiet week for the Drug market, with but few price fluctuations of special importance. An improvement in business cannot be very remote in view of the fact that the chief consuming season has almost begun. To some extent the inactivity of the Drug market can no doubt be attributed to the mildness of the weather, but this does not account entirely for the slowness of the demand. Cod-liver oil continues very quiet and prices have a downward tendency; the new Norwegian fishings will be commencing before long, and the trend of prices will then depend upon the results of the fishings. Importers of quicksilver have made a small advance in the price of the metal, but at present the quotations for calomel, corrosive sublimate, and other mercurials are unchanged. The value of opium is well maintained, and in the primary markets there is an upward tendency and a fair business has been done. Menthol is quiet and inclined towards lower prices. Ipecacuanha tends dearer. Prospective buyers of essence of lemon are anticipating lower prices in due course.

